# Improved Reliability of Electromyography-Based Neuromuscular Monitoring During Laparoscopic Surgery Achieved by the Modified Attachment Method for Nihon-Kohden NM-345Y™ Stimulating Electrodes: A Case Report

**DOI:** 10.7759/cureus.54024

**Published:** 2024-02-11

**Authors:** Shohei Kaneko, Madoka Makino, Kana Miyagawa, Hiroaki Murata, Tetsuya Hara

**Affiliations:** 1 Department of Anesthesiology and Intensive Care Medicine, Nagasaki University Graduate School of Biomedical Sciences, Nagasaki, JPN

**Keywords:** monitoring accuracy, forearm position, stimulating electrode attachment, electromyography, neuromuscular monitoring, neuromuscular blocking agents

## Abstract

Neuromuscular monitoring is crucial during the administration of neuromuscular blocking agents owing to individual variations in their effects. In electromyography (EMG)-based neuromuscular monitoring using the EMG electrodes (NM-345Y™, Nihon-Kohden Corporation, Tokyo, Japan) following the manufacturer-recommended attachment method, the accuracy of neuromuscular monitoring may be reduced when forearm limb position is changed. We previously devised a novel attachment method for NM-345Y™ stimulating electrodes in adult volunteers to maintain stable monitoring accuracy despite changes in forearm position. Its effectiveness in clinical practice was evaluated by conducting a descriptive study on a 52-year-old woman undergoing laparoscopic uterine surgery.

NM-345Y™ electrodes were attached to each forearm following the manufacturer’s recommendations (Pattern N) and our novel method (Pattern C). In Pattern C, NM-345Y™ was attached without ultrasound guidance so that the ulnar nerve crossed the line connecting the centers of the anode and cathode of the stimulating electrode. Pattern C exhibited consistent EMG-based monitoring accuracy even with changes in forearm position despite a smaller stimulus current value at calibration. Additionally, Pattern C displayed reliable recovery of the train-of-four (TOF) response after sugammadex administration in the original forearm position, with no observed adverse events. In contrast, Pattern N showed unstable monitoring accuracy after forearm position changes, highlighting the danger of imprecise EMG-based neuromuscular monitoring during the administration of neuromuscular blocking agents.

The study's strength lies in identifying Pattern C, where the ulnar nerve crosses the line connecting the anode and cathode, significantly enhancing monitoring accuracy. This novel attachment method holds promise to improve EMG-based neuromuscular monitoring precision in surgery involving forearm limb position changes, although further research is required to assess its utility comprehensively.

## Introduction

Neuromuscular monitoring should be performed during the administration of neuromuscular blocking agents owing to individual differences in their effects [1]. If neuromuscular blocking agents are administered to patients without neuromuscular monitoring, intraoperative body movements and/or residual postoperative muscle relaxation may occur [2-4]. To avoid these adverse events, it is essential to maintain the necessary extent of intraoperative muscle relaxation and accurately assess muscle recovery before and after tracheal extubation; this can be accomplished by employing neuromuscular monitoring [1]. In addition, clinicians should be aware that sensitivity to neuromuscular blocking agents varies depending on the muscle being monitored [5]. For instance, the adductor pollicis muscle, which contracts upon ulnar nerve stimulation, is more sensitive to the effects of neuromuscular blocking agents compared with the orbicularis oculi or corrugator supercilii muscles, which contract upon facial nerve stimulation. Thus, monitoring targeted to muscles in the ulnar nerve-innervated area may more accurately reflect the recovery of the pharyngeal muscle, the last muscle to recover from the effects of neuromuscular blocking agents.

In electromyography (EMG)-based neuromuscular monitoring using EMG electrodes (NM-345Y™, Nihon-Kohden Corporation, Tokyo, Japan), it is recommended that the EMG electrodes should be attached along the ulnar nerve with the distal ulnar corner of the stimulating electrodes matched to the pisiform bone [6]. However, the traditionally recommended method may decrease the accuracy of EMG-based neuromuscular monitoring when the forearm limb position changes. As a clue to monitoring accuracy deterioration, a previous study reported that the ulnar nerve may be separated from the skin at the stimulating electrode site and out of the stimulation range of the electrode when the forearm supinates or pronates during ulnar nerve stimulation in EMG measurements [7]. Previously, we devised a novel attachment method for NM-345Y™ stimulating electrodes to maintain stable monitoring accuracy despite changes in the forearm position [8]. In this experimental study on healthy adult volunteers, we demonstrated that our novel method resulted in a less affected EMG-based neuromuscular monitoring accuracy by forearm position changes compared with the method recommended by Nihon-Kohden. However, the clinical implications of our attachment method have not been elucidated. Therefore, we evaluated the effectiveness of our method by comparing monitoring accuracy in a patient undergoing laparoscopic surgery under general anesthesia, with NM-345Y™ electrodes attached, using two different attachment methods simultaneously.

The patient provided written informed consent for the publication of this study. The study received approval from the Institutional Review Board of Nagasaki University Hospital, Nagasaki, Japan (approval number: 23101634). This article was previously presented as a meeting abstract at the 27^th ^Annual Meeting of the Japanese Society for Neuroscience in Anesthesiology and Critical Care on May 20, 2023.

## Case presentation

**Figure 1:**
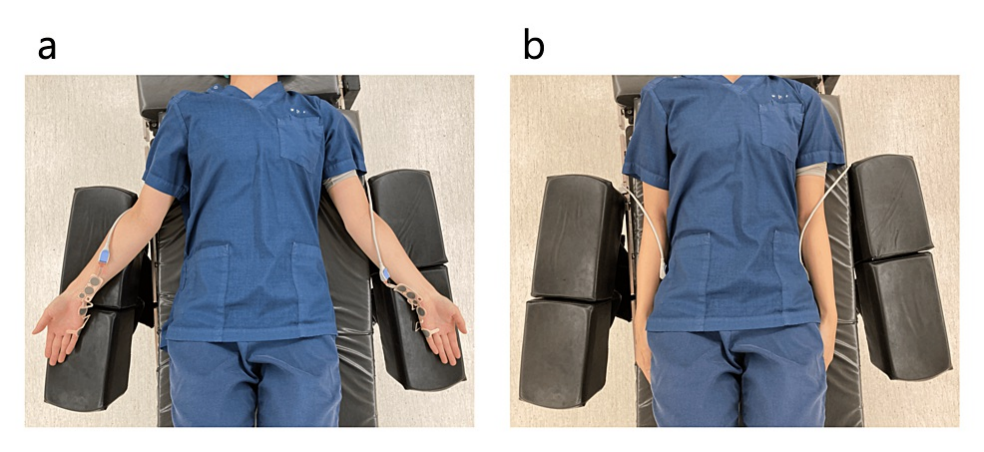
Two forearm limb positions (a) Forearms in 90-degree supination; (b) Forearms in 0-degree pronation

**Figure 2:**
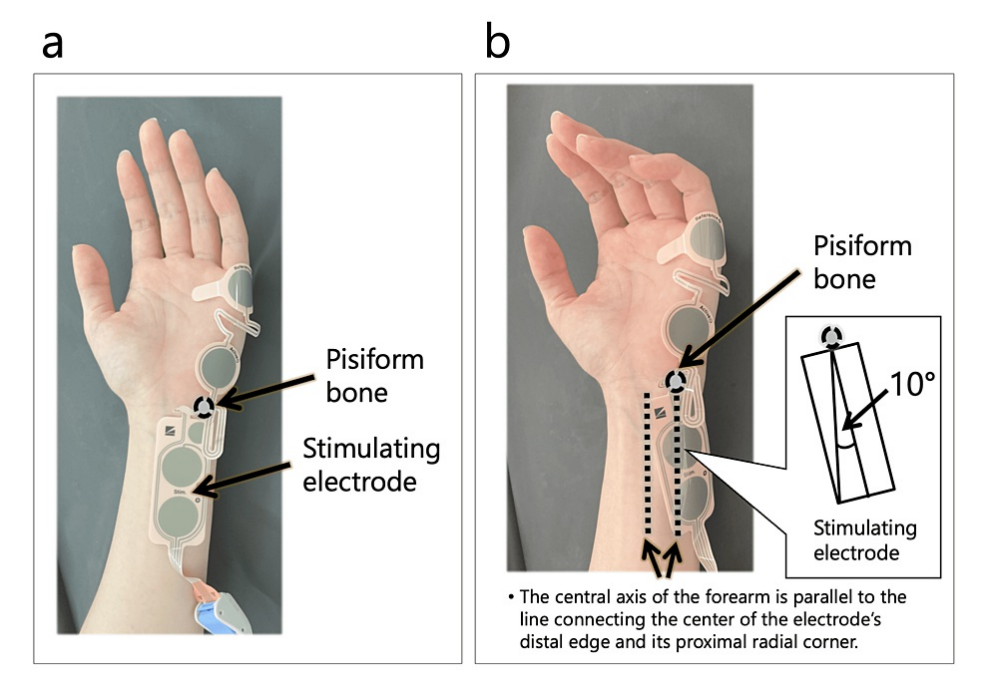
Electromyography electrodes NM-345Y™ and the two attachment patterns (a) Pattern N; (b) Pattern C

A 52-year-old woman (height: 162 cm; weight: 52 kg) with no note complications was scheduled to undergo laparoscopic uterine cancer surgery. Before general anesthesia induction, the patient was placed in a horizontal supine position with the forearms in 90-degree supination (Figure 1). Two NM-345Y™ were attached to the patient’s left and right forearms, each using different attachment methods (Figure 2) [8]. One NM-345Y™ was attached to the patient’s left forearm using the method recommended by Nihon-Kohden (Pattern N) as described in the instruction manual of the electromyographic module (AF-201P™, Nihon-Kohden Corporation, Japan, revised on June 6, 2022). The other NM-345Y™ was attached to the patient’s right forearm, ensuring that the ulnar nerve crossed the line connecting the centers of the anode and cathode of the stimulating electrode without ultrasound guidance (Pattern C). Consequently, the anode of the stimulating electrodes deviated approximately 10 degrees to the ulnar side in Pattern C. Each NM-345Y™ was connected to an individual AF-201P™.

After inducing anesthesia with thiamylal and remifentanil, calibration was performed simultaneously on both forearms. Stimulus current values and sensitivity at calibration were 30 mA and 9.48 mV for Pattern N and 21 mA and 13.8 mV for Pattern C, respectively. The patient received 40 mg of rocuronium following initial train-of-four (TOF) measurements. The trachea was intubated 3 min after the rocuronium administration when the TOF count reached zero in both patterns.

**Figure 3:**
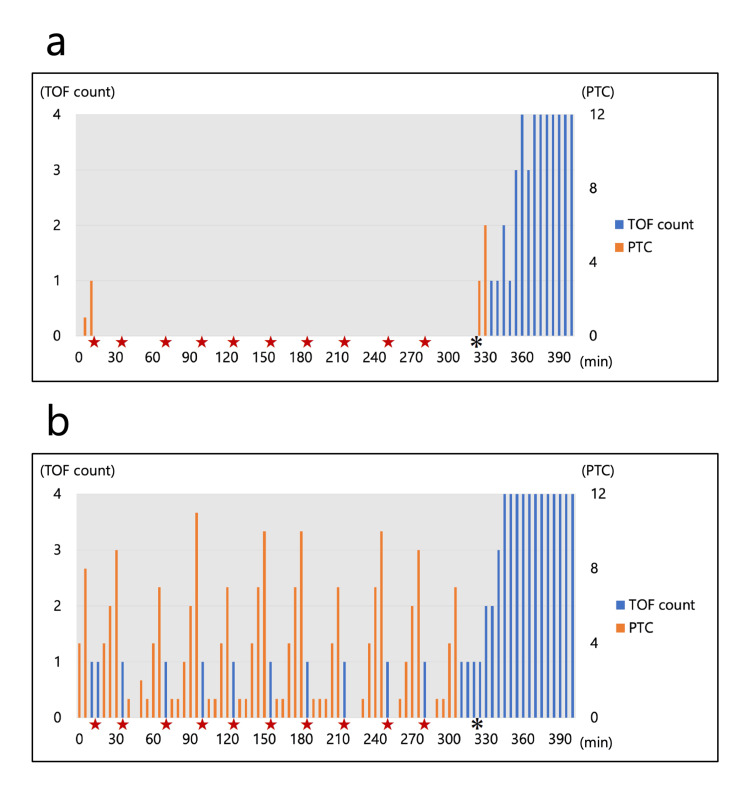
TOF and PTC after forearm position change into 0-degree pronation (a) Pattern N; (b) Pattern C The bars in the graph indicate the TOF count and PTC. The horizontal axis indicates the time course every 5 min after the forearm position changes into 0-degree pronation. The star symbols indicate when 10 mg of rocuronium was administered. The asterisk indicates when the forearm position was changed into 90-degree supination after surgery. TOF, train-of-four; PTC, post-tetanic count

After completing anesthesia induction, the patient was repositioned into the open leg position with both forearms in 0-degree pronation (Figure 1). Anesthesia was maintained using sevoflurane, remifentanil, and rocuronium. The TOF count and post-tetanic count (PTC) were simultaneously measured in both forearms every 15 s and 5 min, respectively, during anesthesia. A bolus dose of rocuronium 10 mg was administered if a TOF count of one persisted for more than 1 min in either forearm during surgery. The TOF count and PTC in both patterns after the forearm position change into 0-degree pronation are presented in Figure 3. In Pattern C, the TOF count and PTC disappeared after rocuronium bolus administration and recovered over time, even after the forearm position change. However, TOF count and PTC were difficult to measure after the forearm position changed in Pattern N, although the patient should have recovered from the neuromuscular blocking state.

We observed no difficulty in securing the surgical field during laparoscopic manipulation, nor subcutaneous emphysema occurrence. In Pattern N, the TOF count and PTC could be measured after changing the forearm position into 90-degree supination after surgery. Upon awakening from anesthesia, the TOF count was four for both patterns. When the TOF ratio reached 45% in Pattern N and 69% in Pattern C, the patient was administered 100 mg of sugammadex. After 5 min, the TOF ratios were 83% and 99% for Patterns N and Pattern C, respectively, and the trachea was extubated. Although the TOF ratio in Pattern N at 5 min after sugammadex administration was below 90%, which is a general extubation criterion in EMG-based neuromuscular monitoring [5], no neuromuscular blocking symptoms were observed after extubation.

## Discussion

Our investigation into EMG-based neuromuscular monitoring during laparoscopic surgery with NM-345Y™ electrodes revealed distinct differences between Pattern N and Pattern C. Notably, Pattern C exhibited superior stability in EMG-based neuromuscular monitoring accuracy even when the forearm position changed, although the stimulus current value at calibration was smaller in Pattern C than in Pattern N. This result supports the fact that in Pattern C the stimulus current value at calibration was sufficient to stimulate the ulnar nerve even during forearm pronation. In addition, the recovery of the TOF response after sugammadex administration in Pattern C was also reliable, and no adverse events potentially indicative of residual muscle relaxation occurred.

Administrating neuromuscular blocking agents under imprecise neuromuscular monitoring is dangerous because it may lead to intraoperative body movements and/or residual postoperative muscle relaxation [2]. In the present case, EMG-based neuromuscular monitoring using Pattern N struggled to measure TOF counts and PTC after the forearm position changed into 0-degree pronation. Furthermore, although TOF and PTC appeared in Pattern N after the forearm position was returned to 90-degree supination postoperatively, the trend of their appearance did not fully reflect recovery from the state of muscle relaxation. For example, TOF counts increased from one to two without additional neuromuscular blocking agent administration but then returned to one again. These findings indicate that EMG-based neuromuscular monitoring using Pattern N was inaccurate not only after changing the forearm position into 0-degree pronation but also after returning to the original forearm position.

The strength of our study lies in the identification and demonstration of Pattern C, which significantly enhances the stability of EMG-based neuromuscular monitoring accuracy during laparoscopic surgery. The robustness of this finding is evident in the consistent TOF count and PTC measurements even when the forearm position changed, suggesting the potential clinical significance of Pattern C in overcoming the limitations associated with Pattern N. However, this study involved a single patient undergoing laparoscopic uterine cancer surgery, limiting the generalizability of the findings. Further investigations with larger and more diverse patient cohorts are needed to validate the broader applicability of Pattern C.

## Conclusions

A novel attachment method, wherein the ulnar nerve crosses the line connecting the centers of the anode and cathode of the NM-345Y™ stimulating electrode, may stabilize the EMG-based neuromuscular monitoring accuracy irrespective of forearm position. The observed strength of the novel attachment method in maintaining reliable monitoring outcomes and its practical application in a clinical scenario suggests a promising avenue to improve the precision of EMG-based neuromuscular monitoring. However, further research is required to evaluate the utility of this method.
